# Identifying risk factors associated with refractoriness to radioiodine therapy in differentiated thyroid cancer

**DOI:** 10.20945/2359-4292-2025-0032

**Published:** 2025-08-18

**Authors:** Fernando Barros Costa Ribeiro, Ana Gregória Ferreira Pereira de Almeida, Adriana de Sá Caldas, Gilvan Cortês Nascimento, Rossana Santiago de Sousa Azulay, Conceição de Maria Ribeiro Veiga Parente, Manuel dos Santos Faria, Marcelo Magalhães, Italo Campinho Braga de Araujo Lima, Carla Souza Pereira Sobral

**Affiliations:** 1 Departamento de Endocrinologia e Metabologia, Hospital Universitário, Universidade Federal do Maranhão, São Luís, Maranhão, Brasil; 2 Grupo de Pesquisa Clínica e Molecular em Endocrinologia e Metabologia, Hospital Universitário, Universidade Federal do Maranhão, São Luís, Maranhão, Brasil

**Keywords:** Differentiated thyroid carcinoma, Refractory radioiodine, Possible associated factors

## Abstract

**Objective:**

To identify factors potentially associated with radioiodine-refractory disease among patients treated for differentiated thyroid carcinoma at a referral center in Northeastern Brazil.

**Methods:**

A total of 554 medical records of patients with differentiated thyroid carcinoma treated between January 2010 and August 2024 were evaluated. Radioiodine-refractory disease tumors were detected in 44 (7.9%) patients. Clinical, laboratory, and radiological data were compared between the radioiodine-refractory disease and non-radioiodine-refractory disease groups to determine factors associated with poor differentiated thyroid carcinoma outcomes.

**Results:**

Factors most strongly associated with progression to radioiodine-refractory disease included older age, increased number of surgeries performed, aggressive histological subtypes, larger tumor size, vascular invasion, extrathyroidal extension, compromised margins, lymph node metastasis, distant metastasis at diagnosis and during follow-up, higher malignant tumor classification staging, high risk of recurrence, high thyroglobulin levels prior to radioiodine therapy, higher doses and greater number of radioiodine therapy doses, and higher frequency of incomplete responses within the first year post-treatment.

**Conclusion:**

Identifying possible factors associated with radioiodine-refractory disease development may allow early diagnosis and a more effective treatment.

## INTRODUCTION

Thyroid cancer (TC) is the most prevalent endocrine neoplasia (^1^), and its global incidence has increased significantly in recent decades (^2^). In Brazil, its incidence ranges from 11 to 15 cases per 100,000 individuals, ranking as the fifth most frequent malignant neoplasm in women (^3^). Histopathologically, TC is classified into three main types: differentiated thyroid carcinoma (DTC), medullary thyroid carcinoma, and anaplastic thyroid carcinoma (^4^). The DTC type represents approximately 90% of all TC cases and includes papillary thyroid carcinoma (84%), follicular thyroid carcinoma (4%) and oncocytic carcinoma (3%) (^5^).

The standard treatment for DTC is surgery, with or without subsequent radioiodine therapy (RAI) (^6^). During follow-up, local recurrence occurs in up to 20% of cases, while distant metastases develop in 10% within the first 10 years (^7^). Therapeutic approaches for such cases include RAI, surgical resection of metastases, and radiotherapy (RT) (^7^). However, 5 to 15% of DTCs are refractory to RAI treatment, presenting a poor prognosis and a 10-year survival rate of only 10% (^8^). The most recent guidelines and studies for managing CT have defined the following criteria for tumors refractory to iodine: patients with tumors that do not show uptake on scintigraphy after the initial RAI therapy; patients with more than one metastatic lesion, with at least one target lesion lacking RAI uptake on the post-iodine examination; patients whose tumors demonstrated structural progression soon after RAI therapy, despite showing uptake on the post-iodine scan (12 to 16 months after treatment); and patients who underwent a cumulative dose of 600 mCi or more of RAI without any sign of remission (^9-12^). Determining whether a patient has radioiodine-refractory disease (RAIR) requires comprehensive treatment, given the limitations of the classifications and the need for individualized patient care (^13^). Notably, no current definition offers an absolute confirmation of RAIR (^14^).

This study aimed to identify factors potentially associated with RAIR among patients treated for differentiated TC at a referral center in Northeastern Brazil. The specific objectives were: to analyze the clinical, epidemiological, and laboratory profiles of patients with RAIR DTC; to describe mortality risk stratification, risk of disease recurrence, and time to refractoriness confirmation; and to evaluate the use of multikinase inhibitors (MKI).

## METHODS

This retrospective cohort study was based on the analysis of medical records of patients diagnosed with DTC treated at the *Departamento de Endocrinologia e Metabologia* of the *Hospital Universitário* of the *Universidade Federal do Maranhão* from January 2010 to August 2024.

**Figure 1:**
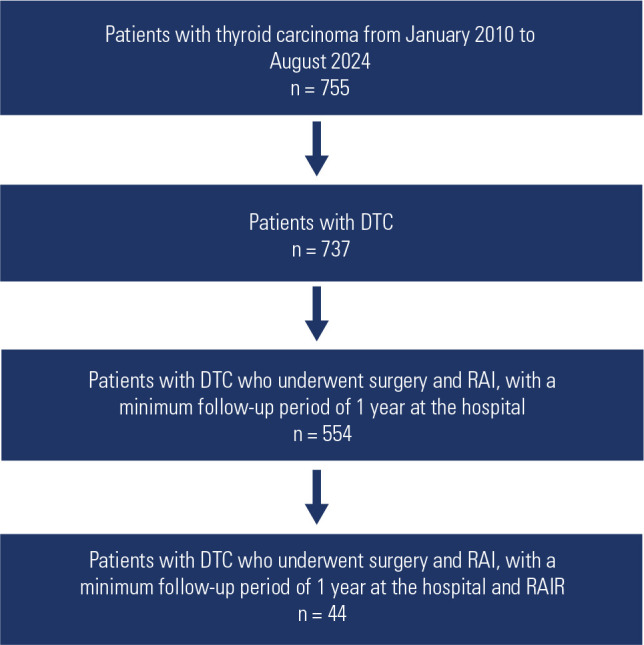
Patients with thyroid carcinoma.

DTC: differentiated thyroid carcinoma; RAI: radioiodine therapy; RAIR: radioiodine-refractory disease.

The inclusion criteria comprised patients with DTC who underwent surgery and RAI, with a minimum follow-up period of 1 year at the hospital. Patients who did not undergo RAI or were diagnosed with medullary thyroid carcinoma or anaplastic thyroid carcinoma were excluded (**Figure 1**). The criteria used to define RAIR were: absence of RAI uptake on scintigraphy following initial RAI therapy; multiple metastatic lesions, with at least one without RAI uptake in the post-iodine examination; structural disease progression shortly after RAI therapy despite having uptake (typically within 12 to 16 months); and lack of remission following a cumulative dose of 600 mCi or more (^9-12^).

Data collection was performed by filling out a standardized form that included demographic, clinical, and laboratory data: sex, age at CT diagnosis, follow-up duration, type and number of surgeries performed, anatomopathological characteristics, I131 dosage and frequency, pre-RAI thyroglobulin (TG) levels, Classification of Malignant Tumors (TNM), risk of recurrence, dynamic risk stratification in the first year, distant metastasis, use of positron emission tomography (PET), time to RAIR determination and the criterion used, treatments with radiotherapy (RT) and bisphosphonate, and use of MKI. In the TNM classification system established by the American Joint Committee on Cancer (AJCC), 8th edition (^15^).

Statistical analyses were tabulated in Excel (v. 2019, Microsoft Office, USA) and analyzed in Statistical Package for the Social Sciences (SPSS, IBM, USA), version 26. Categorical variables were expressed as absolute frequencies (n) and percentages (%), while numerical variables were reported as median, minimum, and maximum values. Normality was verified using the Shapiro-Wilk test. The Mann-Whitney U test was used to compare numerical variables according to the RAIR criteria, and Fisher’s exact test was used for associations between numerical and categorical variables. All statistical associations were considered statically significant at p < 0.05.

This study was approved by the Research Ethics Committee (CAAE: 79376724.8.0000.5086). Participant confidentiality was strictly maintained, and all participants consented via the Free and Informed Consent Form.

## RESULTS

Of the 554 patients with DTC, 44 (7.9%) were diagnosed with RAIR. The majority were female (n = 40; 91%). The median age at DTC diagnosis was 50 years (9 to 83), and the median follow-up duration was 8.5 years (2 to 31) (**Table 1**).

**Table 1:** Characteristics of patients with radioiodine-refractory disease differentiated thyroid carcinoma (n = 44)

Category	
Age at diagnosis, year	
Median (minimum-maximum)	50 (9-83)
< 55	24 (54.5)
≥ 55	20 (45.5)
Follow-up duration, year, median (minimum-maximum)	8.5 (2-31)
Sex	
Male	4 (9)
Female	40 (91)
Histology	
Papillary	39 (88.6)
Classical subtype	24 (54.5)
Follicular subtype	11 (25)
Diffuse sclerosing subtype	1 (2.3)
Tall cell subtype	2 (4.5)
Hobnail subtype	1 (2.3)
Follicular	3 (6.9)
Oncocytic	1 (2.3)
Not described	1 (2.3)
Tumor size, cm, median (minimum-maximum)	3.1 (0.1-7.0)
Microcarcinoma	1 (2.3)
1-4	23 (52.3)
> 4	9 (20.5)
Not described	7 (15.9)
Multifocality	
Absent	32 (72.7)
Present	12 (27.3)
Extrathyroidal extension	
Absent	15 (34.1)
Present	19 (43.2)
Not described	10 (22.7)
Margin commitment	
Not committed	20 (45.5)
Committed	14 (31.8)
Not described	10 (22.7)
Lymph nodes involved during follow-up	
Not committed	9 (20.5)
Committed	35 (79.5)
< 10	20 (45.5)
≥ 10	12 (27.3)
Not described	3 (6.8)
Vascular invasion	
Absent	17 (38.6)
Present	22 (50)
Not described	5 (11.4)
Stimulated TG pre-therapeutic iodine dose (before first dose) ng/mL, median (minimum-maximum)	66.7 (6.1-517)
< 10	1 (2.3)
> 10	19 (43.2)
Not described	24 (54.5)
Total dose of I131, mCi, median (minimum-maximum)	400 (130-650)
Number of iodine doses	
1	4 (9.1)
2	21 (47.7)
3	15 (34.1)
4	4 (9.1)
TNM staging at diagnosis	
I	25 (56.8)
II	10 (22.7)
III	3 (6.8)
IVa	1 (2.3)
IVb	1 (2.3)
Risk of recurrence	
Low	3 (6.8)
Intermediary	11 (25)
High	26 (59.1)
Not described	4 (9.1)
Dynamic staging in the first year	
Excellent response	0
Incomplete biochemical response	17 (38.6)
Incomplete structural response	14 (31.8)
Undetermined response	3 (6.8)
Not described	10 (22.7)
Distant metastasis in follow-up	
Present	27 (61.4)
Lung	20 (74.1)
Bone	1 (3.7)
Lung and bone	4 (14.8)
Lung, bone, and brain	2 (7.4)
Absent	17 (38.6)
Distant metastasis at diagnosis	
Absent	30 (68.2)
Present	12 (27.3)
Not described	2 (4.5)

Results expressed as n (%) if not described in a different way.

TG: thyroglobulin; TNM: Classification of Malignant Tumors.

The most common histological subtype was papillary thyroid carcinoma, identified in 39 patients (88.6%). The median tumor size was 3.1 cm (0.1 to 7.0 range). Lymph node metastases occurred in 35 patients (79.5%) during follow-up, with 20 (57.1%) having fewer than 10 affected nodes. Surgical margin involvement was found in 14 patients (31.8%). Multifocality was present in 12 patients (27.3%), and extrathyroidal extension was observed in 19 patients (43.2%) (**Table 2**).

**Table 2:** Assessment of radioiodine-refractory disease criteria of patients with differentiated thyroid carcinoma

Refractoriness criteria	
Multiple metastatic lesions, with at least one without RAI uptake in the post-iodine examination	9 (20.5)
Structural disease progression shortly after RAI therapy despite having uptake	5 (11.4)
Lack of remission following a cumulative dose of 600 mCi or more	7 (15.9)
Absence of RAI uptake on scintigraphy following initial RAI therapy	23 (52.3)

Results expressed as n (%).

RAI: radioiodine therapy.

Postoperative evaluation showed a median pre-RAI stimulated TG level of 66.7 ng/mL (6.1 to 517 range) (**Table 3**).

**Table 3:** Clinical profile and refractoriness of patients with differentiated thyroid carcinoma

Refractoriness	Yes		No	p-value^*^
Sex				
Female	40 (90.9)		476 (93.3)	0.54
Male	4 (9.1)		34 (6.7)	
Age at diagnosis, years	50 (9-83)		44 (6-77)	< 0.01^†^
Follow-up time, years	8.5 (2-31)		11.0 (1-29)	0.58^†^
Number of surgeries				
1	19 (43.2)		335 (65.7)	< 0.01
> 1	25 (56.8)		175 (34.3)	
Histology				
Papillary	39 (90.7)		399 (78.7)	0.31
Follicular	3 (7.0)		81 (16.0)	
Oncocytic	1 (2.3)		22 (4.3)	
Papillary histological subtype				
Classical	24 (61.5)		269 (67.6)	< 0.01
Follicular	11 (28.2)		119 (29.9)	
Tall cell	2 (5.1)		3 (0.8)	
Hobnail	1 (2.6)		0 (0.0)	
Diffuse sclerosing	1 (2.6)		1 (0.3)	
Others^‡^	0 (0.0)		6 (1.7)	
Tumor size, cm	3.1 (0.1-7.0)		2.0 (0.1-8.0)	< 0.01†

Results expressed as n (%) or median (minimum-maximum).

* Fisher’s exact;

† Mann-Whitney;

‡ solid/trabecular, columnar, clear cell and cribriform-morular.

In the TNM classification system 25 patients (56.8%) were diagnosed at stage I. In contrast, 26 patients (59.1%) were classified as high risk for occurrence (**Table 4**). During the first year after surgery, dynamic risk stratification identified an incomplete biochemical response in 17 patients (38.6%), an incomplete structural response in 14 (31.8%), and an indeterminate response in 3 (6.8%). Distant metastasis was detected in 27 patients (61.4%) during follow-up, with 12 of these cases (27.3%) already present at the time of DTC diagnosis. All RAIR patients received RAI therapy, with a median cumulative dose of 400 mCi (130 to 650 range), administered in up to four sessions during follow-up. Total thyroidectomy was performed in all patients, and 26 (59.1%) underwent more than one surgery. Neck dissection was required in 38 patients (86.4%) over the follow-up. Additionally, nine patients (20.5%) received radiotherapy, and 6 (13.6%) underwent bisphosphonate therapy for bone metastases.

**Table 4:** Tumor characteristics and refractoriness of patients with differentiated thyroid carcinoma

Refractoriness	Yes	No	p-value^*^
Multifocality			
Yes	12 (27.3)	136 (26.7)	0.58
No	32 (72.7)	373 (73.3)	
Vascular invasion			
Yes	22 (56.4)	90 (27.7)	< 0.01
No	17 (43.6)	235 (72.3)	
Extrathyroidal extension			
Yes	19 (55.9)	121 (36.2)	0.02
No	15 (44.1)	213 (63.8)	
Margin commitment			
Yes	14 (40.0)	49 (14.9)	< 0.01
No	21 (60.0)	279 (85.1)	
Lymph nodes involved during follow-up			
Yes	35 (79.5)	80 (15.7)	< 0.01
No	9 (20.5)	430 (84.3)	
Number of lymph nodes affected	7 (1-35)	3 (1-16)	< 0.01†
Tumor size			
T1a	2 (5.1)	106 (22.6)	< 0.01
T1b	2 (5.1)	81 (17.3)	
T2	9 (23.1)	93 (20.1)	
T3a	8 (20.5)	79 (16.9)	
T3b	11 (28.2)	87 (18.6)	
T4a	7 (17.9)	21 (4.5)	
Lymph node involvement at diagnosis			
N1a	5 (11.9)	85 (17.2)	< 0.01
N1b	24 (57.1)	25 (5.1)	
N0	13 (31.0)	383 (77.7)	
Distant metastasis at diagnosis			
M1	12 (28.6)	18 (3.6)	< 0.01
M0	30 (71.4)	476 (96.4)	
Staging at diagnosis			
1	25 (62.5)	427 (86.4)	< 0.01
2	10 (25.0)	62 (12.6)	
3	3 (7.5)	3 (0.6)	
4a	1 (2.5)	0 (0.0)	
4b	1 (2.5)	2 (0.4)	
Risk of recurrence			
Low	3 (7.5)	228 (48.1)	< 0.01
Intermediary	11 (27.5)	157 (33.1)	
High	26 (65.0)	89 (18.8)	

Results expressed as n (%) or median (minimum-maximum).

* Fisher’s exact; †Mann-Whitney.

The median time to RAIR classification was 4.3 years (0.1 to 7.0 range). The most frequent criterion for RAIR diagnosis was radioiodine uptake absence on scintigraphy following the first RAI therapy, observed in 23 patients (52.3%) (**Table 5**).

**Table 5:** Clinical, laboratory, and refractoriness responses of patients with differentiated thyroid carcinoma

Refractoriness	Yes	No	p-value^*^
Stimulated TG pre-iodine dose, ng/mL	66.7 (6.1-517)	6.7 (0.2-505)	< 0.01†
Total dose of I131, mCi	400 (130-650)	100 (30-600)	< 0.01†
Number of doses of I131			
1	4 (9.1)	463 (91.0)	< 0.01
2	21 (47.7)	35 (6.9)	
3	16 (36.4)	6 (1.2)	
4	3 (6.8)	5 (1.0)	
Stratification in the first year			
Biochemistry	17 (50.0)	24 (7.3)	< 0.01
Structural	14 (41.2)	13 (4.0)	
Undetermined	3 (8.8)	86 (26.2)	
Excellent	0 (0.0)	205 (62.5)	
Positron emission tomography			
Abnormal	19 (67.9)	3 (37.5)	0.12
Normal	9 (32.1)	5 (62.5)	
Standardized uptake value, g/mL	4.25 (2.3-20.1)	3.4 (3.3-3.5)	0.24†
Distant metastasis in follow-up			
Yes	27 (61.4)	4 (0.8)	< 0.01
No	17 (38.6)	506 (99.2)	
Location of metastasis			
Lung	20 (74.1)	3 (75.0)	0.88
Bone	1 (3.7)	0 (0.0)	
Lung and bone	4 (14.8)	1 (25.0)	
Lung, bone, and brain	2 (7.4)	0 (0.0)	

Results expressed as n (%) or median (minimum-maximum).

* Fisher’s exact; †Mann-Whitney.

TG: thyroglobulin.

Seven patients (15.9%) began MKI therapy; six (85.7%) received sorafenib, and one (14.3%) lenvatinib. Among patients with DTC, progression to refractoriness was most associated with older age, multiple surgeries, aggressive histological types, larger tumors, vascular invasion, extrathyroidal extension, compromised margins, nodal and distant metastases (at diagnosis and follow-up), advanced TNM stage, high risk of recurrence, elevated pre-RAI TG, higher and more frequent RAI doses, and incomplete response in the first year.

## DISCUSSION

This study evaluated the prevalence and clinical profile of patients with RAIR DTC treated at the *Departamento de Endocrinologia e Metabologia* of the *Hospital Universitário* of the *Universidade Federal do Maranhão* to identify characteristics associated with RAIR compared to the non-RAIR group. The RAIR DTC was identified in 44 patients (7.9%), consistent with the average prevalence in the literature (^8^). Additionally, most RAIR DTC cases were female, aligning with existing data, as well as the median age at diagnosis (^16^). The total thyroidectomy was the initial surgery performed in all patients, and most required additional surgeries during follow-up. These findings match reports of this surgery and cervical dissection in 67% of cases (^17^).

Tumor size, histology, and lymph node metastasis during follow-up reflect findings from previous studies: the most common histological type was the classic papillary, followed by the follicular subtype, and most patients developed lymph node metastasis (^18^). Schubert and cols. (^17^) reported a vascular invasion in 30% of patients, which differed from our study. Conversely, Wassermann and cols. (^19^) found a 51% rate, similar to our findings. Such differences can be attributed to the small sample size and incomplete recording of anatomopathological results in the medical records.

Multifocality was observed in a minority of patients, in line with prior studies: 33% in Schubert and cols. (^17^) and 37.5% in Bel Lakhdar and cols. (^16^). The extrathyroidal extension found by Parvathareddy and cols. (^18^) was 61.2%; 43% in Schubert and cols. (^17^) and 37.5% in Bel Lakhdar and cols. (^16^), with Schubert and cols. (^17^) presenting a closer result to our study. These variations likely stem from the heterogeneity of RAIR disease, small sample sizes, and incomplete anatomopathological data in the medical records. Surgical margin involvement was seen in a minority of patients, present in 35.1% in Shobab and cols. (^20^) and in our findings.

In the initial TNM classification, following the first surgery, the pT3bN1bM0 staging predominated. Tumor staging aligned with the literature, in which T3 was the most prevalent (^19^). However, it was discrepant with lymph node involvement (N1), which was observed in 45% of patients at diagnosis (^19^). Lymph node metastasis was observed at diagnosis in most patients, suggesting a more aggressive disease. As for distant metastasis, only 40% were presented at diagnosis, consistent with the literature (^19^). The predominant staging at diagnosis by the TNM classification was I, aligning with Parvathareddy and cols. (^18^), which reported a 69.4% occurrence. This result can be explained by the predominant age range of the sample (< 55 years) and the small number of distant metastases at diagnosis.

The American Thyroid Association (ATA) classification suggests most patients in this study were at high risk. Hassan and cols. (^21^) reported 26%, contrasting our findings. Lorusso and cols. (^22^) found 48.4%. This difference between studies may indicate the divergence in applying the recurrence risk criteria between institutions and studies, such as using pre-dose TG in risk stratification (^20^,^23^,^24^).

Pre-iodine dose TG values were higher than those in the literature, which reported a median of 56 ng/mL (^19^). The TG is a key biochemical marker for evaluating DTC. In advanced carcinomas, RAIR is not always accurately assessed, as some tumors lose characteristics present in normal follicular cells (dedifferentiation) and stop producing TG (^19^). Most patients were diagnosed with locoregional disease, with some showing distant metastasis, contributing to higher TG values.

Patients received a lower dose of I131 than the 518 mCi in Shobab and cols. (^20^) but a higher dose than the 305 mCi in another study (^19^). Most underwent multiple doses, with fixed doses administered as long as the post-dose scintigraphy showed uptake or even RAIR, leading to higher cumulative doses. Wassermann and cols. (^19^) used dosimetry with lower doses for metastatic disease treatment, while Shobab and cols. (^20^) used fixed doses.

In the first year after surgery, dynamic risk stratification predominantly indicated incomplete biochemistry and structural responses, evidencing a more aggressive disease in these patients. No data were found in the literature for comparison. Dynamic risk stratification is a predictive approach for long-term clinical outcomes (^25^).

At diagnosis, distant metastasis was found in a minority of RAIR patients, as demonstrated by Wasserman and cols. (^19^). During follow-up, most patients developed distant metastasis, consistent with the study by Bel Lakhdar and cols. (^16^), who reported that aggressive carcinomas frequently progress to advanced and metastatic stages.

The lung was the most prevalent site for metastasis, as reported by Ibrahim and Busaidy (^26^) and supported by Schubert and cols. (^17^). PET-CT evaluation was performed in most patients, revealing disease in many of them, aligning with existing literature (^19^). The median SUV value of PET-CT was high, comparable to the median value of 5.37 found by Liu and cols. (^27^). This suggests that our patients had aggressive disease. PET-CT using fluoride-2-glucose may predict disease progression in patients with incomplete structural or biochemical response, as its uptake indicates a higher risk of progression (^28^).

A minority of patients underwent RT treatment, and few patients required bisphosphonate therapy due to bone pain resulting from metastasis. Many of our patients presented with surgically resectable locoregional recurrence that did not require RT, while others had inoperable tumors necessitating its use. The limited number of patients with bone metastases did not justify the broader use of bisphosphonate therapy. No data were found in the literature for comparison.

The diagnosis of RAIR was later compared to the study by Wasserman and cols. (^19^), which showed a delay of 3.3 years. Conversely, Schubert and cols. (^17^) found an average delay of 6.8 years between DTC diagnostics and refractoriness. Factors that may have corroborated the later diagnosis of RAIR in this study included the small number of patients, insufficient institutional experience in such cases, incomplete data in the medical records, and challenges in characterizing these criteria in the literature as a novel concept. The RAIR DTC is associated with abnormal function of the sodium iodide symporter and reduced expression of other iodine-handling genes, including TG, thyroperoxidase, and the thyroid-stimulating hormone receptor (^29^).

The primary criterion for classifying RAIR was absence of RAI uptake on scintigraphy following initial RAI therapy. The second most common was multiple metastatic lesions, with at least one without RAI uptake in the post-iodine examination, a pattern also reported by Shobab and cols. (^20^) and Schubert and cols. (^17^). Wasserman and cols. (^19^) identified the latter criterion as the most frequent, while Parvathareddy and cols. (^18^) reported that the most frequent criterion was structural disease progression shortly after RAI therapy, despite initial uptake on the post-iodine examination.

These findings highlight inconsistencies in the predominant classification criteria for refractoriness due to varied sample sizes and heterogeneous disease progression. In addition, its classification is still controversial and not standardized, a reflection of a complex, multifactorial condition with diverse clinical presentations and responses to treating a new diagnosis. The patients in this study presented heterogeneous disease with uptake and non-uptake lesions, progressing to RAIR even when initial RAI concentration was adequate.

Few patients started MKI; among them, nearly all used sorafenib as first-line treatment. Wasserman and cols. (^19^) observed MKI use in one-third of the patients, while Jerkovich and cols. (^30^) identified sorafenib as first-line treatment in 95.5% of the cases, and only 4.5% received levantinib. Therefore, in our sample, MKI was used in a minority of cases, consistent with the literature. The limited use of MKIs may be attributed to the low symptomatology related to metastases present in the group of patients, and may reflect a more indolent clinical course, minimal or slow progression, and prolonged asymptomatic periods, allowing for active surveillance.

Characteristics possibly associated with RAIR DTC were: older age, multiple surgeries, aggressive histological subtypes, larger tumor size, vascular invasion, extrathyroidal extension, compromised margins, lymph node metastasis, distant metastasis at diagnosis and during follow-up, more advanced TNM staging, high risk of recurrence, high pre-RAI TG levels, higher cumulative RAI doses administered, and incomplete response within the first year.

Schubert and cols. (^17^) identified seven factors most frequently associated with RAIR: age ≥ 55 years at diagnosis, vascular invasion, lymph node metastasis at diagnosis, lung metastasis at diagnosis, bone metastasis at diagnosis, recurrence or persistence of lymph node metastases in the neck, and, finally, recurrence or persistence of lung metastasis. Bel Lakhdar and cols. (^16^) associated refractoriness with age ≥ 54 years at diagnosis, primary tumor diameter ≥ 2.9 cm, lymph node metastasis or distant metastasis during follow-up, and oncocytic histological type. Luo and cols. (^31^) observed that the predictive factors were histological subtype, extrathyroidal extension, BRAFV600E (V-raf Murine Sarcoma Viral Oncogene Homolog B1) mutation, and telomerase reverse transcriptase promoter mutation.

Variability in the factors associated with RAIR likely results from differences in study methods, patient populations, and definitions of refractoriness, reflecting the complexity of the disease. Nevertheless, several factors identified in this study, such as age at diagnosis, histological subtype, vascular invasion, lymph node metastasis at diagnosis, distant metastasis at diagnosis, extrathyroidal extension, tumor size, lymph node involvement during follow-up, and metastasis during follow-up, were similar to those reported in the literature.

One limitation of this study is that RAIR is a new concept, which may have impacted the sample size. Additionally, data collection was incomplete, often because patients received initial care at other hospitals before being admitted to the *Hospital Universitário* of the *Universidade Federal do Maranhão*, and some were lost to follow-up. Logistic regression and univariate and multivariate analysis were not conducted due to missing data, inconsistent timing of variables measurement (at diagnosis versus post-refractoriness), and lack of control over the interval between initial diagnosis and outcome diagnosis. Thus, no cause-and-effect relationship between the variables and RAIR can be drawn from this retrospective analysis. Furthermore, there was no molecular study of this population for cases that required MKI.

Fortunately, few patients with DTC develop RAIR. Characteristics of DTC possibly associated with RAIR were age, multiple surgeries, more aggressive histology, larger tumor size, vascular invasion, extrathyroidal extension, compromised margins, greater number of lymph nodes affected, distant metastasis at diagnosis and during follow-up, more advanced TNM staging, high risk of recurrence, high pre-iodine dose TG levels, higher cumulative RAI doses, and greater incomplete response in the first year (biochemical and structural). Identifying these factors may allow early diagnosis and a more effective treatment of these cases.
